# Change of Serum Biomarkers to Post-Thrombolytic Symptomatic Intracranial Hemorrhage in Stroke

**DOI:** 10.3389/fneur.2022.889746

**Published:** 2022-06-02

**Authors:** Yu Cui, Xin-Hong Wang, Yong Zhao, Shao-Yuan Chen, Bao-Ying Sheng, Li-Hua Wang, Hui-Sheng Chen

**Affiliations:** ^1^Department of Neurology, General Hospital of Northern Theatre Command, Shenyang, China; ^2^Department of Neurology, Haicheng Hospital of Traditional Chinese Medicine, Haicheng, China; ^3^Department of Neurology, Chinese People's Liberation Army 321 Hospital, Baicheng, China; ^4^Department of Neurology, Jiamusi University First Affiliated Hospital, Jiamusi, China; ^5^Department of Neurology, The Second Affiliated Hospital of Harbin Medical University, Harbin, China

**Keywords:** alteplase, intravenous thrombolysis, symptomatic intracranial hemorrhage, biomarkers, microarray analysis

## Abstract

**Background:**

Symptomatic intracranial hemorrhage (sICH) is a terrible complication after intravenous alteplase in stroke, and numerous biomarkers have been investigated. However, the change of biomarkers to sICH has not been well determined.

**Aim:**

To investigate the association between the change of biomarkers and sICH.

**Methods:**

This is a prospective cohort study, and patients with sICH within 24 h after thrombolysis were enrolled, while patients without sICH were matched by propensity score matching with a ratio of 1:1. The blood samples were collected before and 24 h after intravenous thrombolysis (IVT), and preset 49 serum biomarkers were measured by microarray analysis. Protein function enrichment analyses were performed to detect the association between the change of biomarkers and sICH.

**Results:**

Of consecutive 358 patients, 7 patients with sICH in 24 h were assigned to the sICH group, while 7 matched patients without any ICH were assigned to the non-sICH group. A total of 9 biomarkers were found to significantly change before vs. after thrombolysis between groups, including increased biomarkers, such as brain-derived neurotrophic factor, C-C motif chemokine ligand (CCL)-24, interleukin (IL)-6, IL-10, IL-18, and vascular endothelial growth factor, and decreased biomarkers, such as CCL-11, intercellular adhesion molecule-1, and IL-7.

**Conclusions:**

This is the first study to identify changes in serum biomarkers in patients with sICH after IVT, and found that 6 neuroinflammatory and 3 neuroprotective biomarkers may be associated with brain injury following post-thrombolytic sICH.

**Clinical Trial Registration:**

https://www.clinicaltrials.gov, identifier: NCT02854592.

## Introduction

Intravenous alteplase is the main effective treatment for acute ischemic stroke (AIS) within 4.5 h after the onset of symptoms (1). Symptomatic intracranial hemorrhage (sICH) is a rare but severe complication after thrombolysis that is closely related to disability and death (2).

Mountains of studies have investigated predictors for post-thrombolytic sICH in stroke, such as clinical, radiological, and laboratory factors (3–5). Although several biomarkers were found to be associated with post-thrombolytic hemorrhagic transformation (6–11), these biomarkers were only detected at admission. To date, however, only one study investigated the change of serum biomarkers after post-thrombolytic sICH (12). Given that changes in biomarkers before and after sICH maybe reflect the secondary brain injury of sICH, the issue needed to be further investigated.

In the INtravenous Thrombolysis REgistry for Chinese Ischemic Stroke within 4.5 h of onset (INTRECIS) (13), 5 centers were pre-designed to consecutively collect blood samples before and 24 h after thrombolysis. In this study, we tried to identify what serum biomarkers change significantly before thrombolysis vs. after sICH, compared with patients without any ICH and investigated the potential interactions by microarray analysis on 49 preset biomarkers.

## Methods

### Study Population and Procedure

From August 2018 to July 2019, patients receiving intravenous thrombolysis (IVT) within 4.5 h after symptoms onset were consecutively screened to collect blood samples before thrombolysis at five pre-set stroke centers in the INTRECIS study. The INTRECIS is a prospective, nationwide, multi-center, and registry study of consecutive adult patients who were eligible for treatment with IVT within 4.5 h of the onset of symptoms, and has been published recently (13). Patients who received a standard dose of alteplase (0.9 mg/kg, maximum 90 mg; manufacturer: Boehringer Ingelheim) within 4.5 h after symptoms onset were included in the study. The exclusion criteria were as follows: (1) patients received urokinase or non-standard dose of alteplase, (2) patients received endovascular intervention, (3) patients lacked complete clinical data, and (4) blood samples were not collected before or 24 h after IVT. According to the occurrence of sICH 24 h after IVT, enrolled patients were divided into two groups: (1) sICH group: patients who had sICH within 24 h after IVT; (2) non-sICH group: patients who did not have any ICH in 24 h after IVT. Furthermore, as described in our previous study (14), propensity score matching was performed between groups with a ratio of 1:1, the caliper of 0.1, and a nearest-neighbor matching strategy, and operated with control factors, such as age, current smoking, alcohol consumption, gender, systolic blood pressure, diastolic blood pressure, symptom onset-to-treatment time, blood glucose, National Institutes of Health Stroke Scale (NIHSS) score at admission, previous use of antiplatelet, and medical history, and the Trial of Org 10172 in Acute Stroke Treatment (TOAST) classification.

The baseline characteristics and clinical data of recruited patients were obtained from the electronic database: age, current smoking, alcohol consumption, gender, systolic blood pressure, diastolic blood pressure, symptom onset-to-treatment time, blood glucose, National Institutes of Health Stroke Scale (NIHSS) score at admission, previous use of antiplatelet, and medical history, and the Trial of Org 10172 in Acute Stroke Treatment (TOAST) classification. All patients underwent a CT scan at admission and 24 h (or when neurological deterioration occurred within 24 h) after IVT, to evaluate the presence of ICH. According to European Cooperative Acute Stroke Study (ECASS)-II study, sICH was defined as an increase of ≥4 on the NIHSS scores caused by ICH within 36 h (15).

### Laboratory Determinations

The methods of laboratory determinations were described in our previous study (14). Briefly, 4 ml of venous blood were collected for measurement before and 24 h after IVT, respectively. The blood samples were centrifuged at 4°C and 1,000 g for 10 min, and then transferred into a cryotube and stored at −80°C. The customized protein microarray analysis from Raybiotech Inc. was used to simultaneously quantify 49 biomarkers in the above blood samples, which were preset based on published data.

### Statistical Analysis

Descriptive statistics were performed to compare variables between the two groups. Continuous variables with normal distribution were described as means and standard deviation (SD). Continuous variables included age, systolic blood pressure, diastolic blood pressure, blood glucose, NIHSS score, symptom onset to thrombolysis time, and detected circulating concentration of biomarkers. The *t*-tests were used to analyze continuous variables with normal distribution. Additionally, categorical variables were described as numbers and proportions. Categorical variables included gender, medical history, and TOAST classification. The Pearson χ^2^ tests were used to analyze categorical variables.

The empirical Bayes-based linear model method was used to analyze the differential expression of biomarkers between different stages of disease with the R package limma. The differential expression was evaluated by adjusting the value of *p* (BH method) based on moderated *t* statistics. In all analyses, differences were considered statistically significant with a *p* < 0.05. The free statistical language R (version 3.10.3) was used for the outcomes and graph in microarray analysis.

## Results

**Figure 1:**
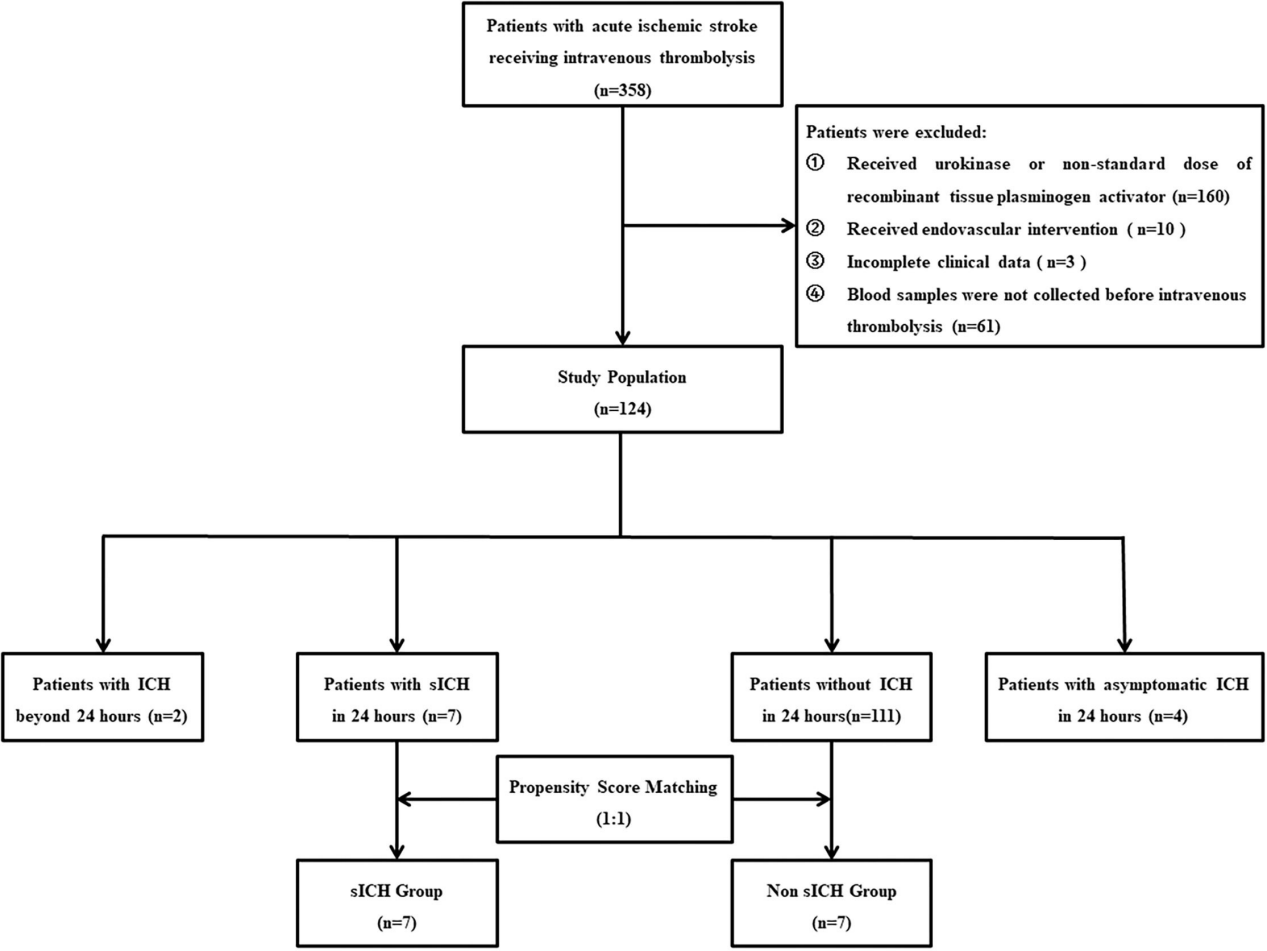
Flow diagram. sICH, symptomatic intracranial hemorrhage.

As shown in Figure 1, 358 patients with thrombolysis were consecutively screened in the present study, and 234 patients were excluded for different reasons: 160 patients received urokinase or the non-standard dose of alteplase, 10 patients received the endovascular intervention, 3 patients with incomplete clinical data, and 61 patients without blood samples collection. Finally, 124 patients were recruited into the current study, including 2 patients with ICH after 24 h, 4 patients with asymptomatic ICH in 24 h, 7 patients with sICH in 24 h, and 111 patients without any ICH in 24 h after IVT. There was no significant difference in the baseline characteristics between included patients and excluded patients (Table 1). With a ratio of 1:1, 7 patients without any ICH and 7 patients with sICH were matched to the non-sICH group and sICH group for final analysis (Figure 1). There was no significant difference in the baseline characteristics between the two groups (Table 2).

**Table 1:** Demographic and baseline characteristics.

**Variables**	**Included (*n* = 124)**	**Excluded (*n* = 234)**	***P*-value**
**Demographics**			
Age, years, mean ± SD	63.8 ± 12.0	64.9 ± 9.6	0.362
Gender, male, *n* (%)	74 (59.7)	156 (66.7)	0.230
Current smoking, *n* (%)	24 (19.4)	65 (27.8)	0.515
Alcohol consumption, *n* (%)	20 (16.1)	47 (20.1)	0.733
**Medical history**, ***n*** **(%)**			
Stroke	12 (9.7)	37 (15.8)	0.257
Hypertension	75 (60.5)	116 (49.6)	0.102
Diabetes mellitus	35 (28.2)	61 (26.1)	0.606
Atrial fibrillation	22 (17.7)	59 (25.2)	0.232
Congestive heart failure	20 (16.1)	30 (12.8)	0.805
Previous use of antiplatelet	13 (10.5)	22 (9.4)	0.913
**Baseline scales, mean** **±SD**			
Systolic blood pressure, mmHg	160.2 ± 23.6	161.2 ± 25.8	0.800
Diastolic blood pressure, mmHg	92.1 ± 14.0	91.1 ± 14.5	0.669
Blood glucose, mmol/L	8.45 ± 3.78	7.42 ± 2.42	0.244
Symptom onset to thrombolysis time, min	174.5 ± 54.5	187.8 ± 64.4	0.757
NIHSS score at admission	5.7 ± 4.8	6.2 ± 5.5	0.521
**TOAST classification**, ***n*** **(%)**			0.063
LAA	46 (37.4)	108 (46.2)	
SAO	17 (13.8)	27 (11.6)	
CE	14 (11.4)	42 (17.9)	
UND	46 (37.4)	57 (24.3)	

*CE, cardiogenic embolism; LAA, large artery atherosclerosis; NIHSS, National Institute of Health Stroke Scale; SAO, small artery occlusion; SD, standard deviation; sICH, symptomatic intracranial hemorrhage; TOAST, the Trial of Org 10172 in Acute Stroke Treatment; UND, undetermined cause*.

**Table 2:** Demographic and baseline characteristics for matched patients.

**Variables**	**sICH (*n* = 7)**	**Non-sICH (*n* = 7)**	***P*-value**
**Demographics**			
Age, years, mean ± SD	64.3 ± 12.3	66.1 ± 9.6	0.757
Gender, male, *n* (%)	3 (42.9)	3 (42.9)	1.000
Current smoking, *n* (%)	1 (14.3)	2 (28.6)	0.515
Alcohol consumption, *n* (%)	1 (14.3)	1 (14.3)	1.000
**Medical history**, ***n*** **(%)**			
Stroke	2 (28.6)	2 (28.6)	1.000
Hypertension	6 (85.7)	5 (71.4)	0.515
Diabetes mellitus	1 (14.3)	2 (28.6)	0.515
Atrial fibrillation	0 (0.0)	1 (14.3)	0.299
Congestive heart failure	3 (42.9)	4 (57.1)	0.593
Previous use of antiplatelet	1 (12.5)	1 (12.5)	1.000
**Baseline scales, mean** **±SD**			
Systolic blood pressure, mmHg	160.0 ± 18.8	146.7 ± 8.7	0.115
Diastolic blood pressure, mmHg	91.0 ± 4.8	86.7 ± 11.8	0.390
Blood glucose, mmol/L	8.29 ± 3.03	7.40 ± 2.26	0.547
Symptom onset to thrombolysis time, min	175.0 ± 66.5	177.6 ± 60.0	0.937
NIHSS score at admission	6.9 ± 6.1	4.7 ± 6.0	0.521
**TOAST classification**, ***n*** **(%)**			0.494
LAA	3 (42.9)	2 (28.6)	
SAO	1 (14.3)	1 (14.3)	
CE	0 (0.0)	2 (28.6)	
UND	3 (42.9)	2 (28.6)	

*CE, cardiogenic embolism; LAA, large artery atherosclerosis; NIHSS, National Institute of Health Stroke Scale; SAO, small artery occlusion; SD, standard deviation; sICH, symptomatic intracranial hemorrhage; TOAST, the Trial of Org 10172 in Acute Stroke Treatment; UND, undetermined cause*.

### Changes in Biomarkers After sICH

**Figure 2:**
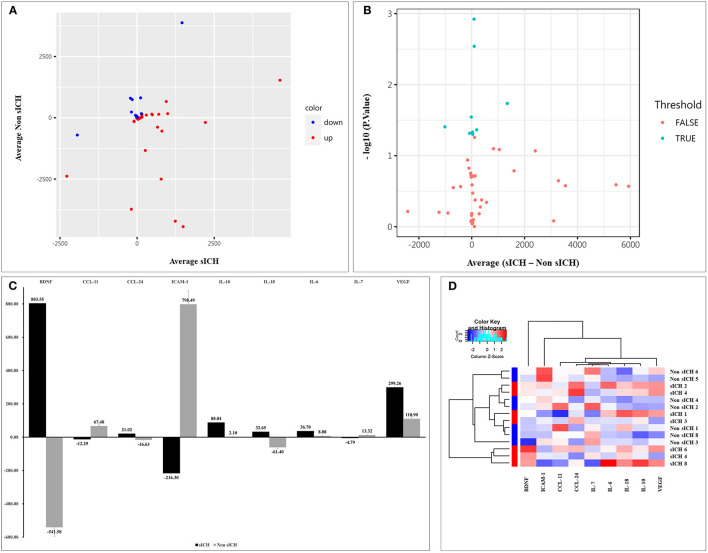
Results of detected biomarkers in the microarray analysis. **(A)** The scatter plot for detected biomarkers; the *X*-axis represents the average of serum levels in the sICH group, while the *Y*-axis represents the average of serum levels in the non-sICH group; compared with the non-sICH group, the blue point represents biomarkers with decrease in the sICH group, while the red point represents biomarkers with increase. sICH, symptomatic intracranial hemorrhage. **(B)** The volcano plot for detected biomarkers; the *X*-axis represents the average of change in serum levels between two groups while the *Y*-axis represents the –log10 *p*; the cyan point represents the biomarkers with a significant difference, while the red point represents the biomarkers without significant difference. sICH, symptomatic intracranial hemorrhage. **(C)** The column plot for identified biomarkers; the *X*-axis represents the identified biomarkers, while the *Y*-axis represents the average of serum levels in two groups; the deep color represents the sICH group, while the light color represents the non-sICH group. **(D)** The heatmap for identified biomarkers; the red color represents biomarkers with an increase, while the blue color represents the biomarkers with decrease; the darker the color, the more significant the difference of biomarkers. BDNF, brain-derived neurotrophic factor; CCL-11, C-C motif chemokine ligand (CCL)-11; CCL-24, C-C motif chemokine ligand (CCL)-24; ICAM-1, intercellular adhesion molecule-1; IL-10, interleukin-10; IL-18, interleukin-18; IL-6, interleukin-6; IL-7, interleukin-7; VEGF, vascular endothelial growth factor; and sICH, symptomatic intracranial hemorrhage.

Compared with the non-sICH group, significant changes of 9 biomarkers before and after sICH were observed in the sICH group (*p* < 0.05), including a significant increase in brain-derived neurotrophic factor (BDNF), C-C motif chemokine ligand (CCL)-24, interleukin (IL)-6, IL-10, IL-18, and vascular endothelial growth factor (VEGF), and a significant decrease in IL-7, intercellular adhesion molecule (ICAM)-1, and CCL-11 (Table 3). The scatter and volcano plot showed results of all measured biomarkers (Figures 2A,B). The column plot and heatmap showed the results of the identified biomarkers (Figures 2C,D).

**Table 3:** Detected circulating concentration of identified biomarkers.

**Biomarkers**	**sICH (*****n*** **=** **7)**		*P*-value*	**Non-sICH (*****n*** **=** **7)**		*P*-value*	***P*-value^†^**
	**Pre-IVT**	**Post-IVT**		**Pre-IVT**	**Post-IVT**		
**Mean** **±SD, pg/ml**							
**Up regulated**							
BDNF	2,839.13 ± 1,189.63	3,642.68 ± 657.55	0.144	3,713.66 ± 1,221.32	3,060.08 ± 1,239.81	0.340	0.018
CCL-24	175.24 ± 54.07	196.26 ± 43.92	0.440	215.51 ± 55.26	203.20 ± 58.11	0.692	0.049
IL-6	6.68 ± 7.84	43.38 ± 28.99	0.015	1.64 ± 3.41	9.51 ± 6.49	0.015	0.046
IL-10	63.06 ± 27.68	151.10 ± 57.46	0.006	44.25 ± 27.42	44.79 ± 10.56	0.963	0.001
IL-18	200.01 ± 46.90	232.66 ± 80.81	0.373	307.30 ± 108.35	272.63 ± 62.28	0.477	0.003
VEGF	326.26 ± 192.82	625.52 ± 200.75	0.015	280.08 ± 171.71	420.51 ± 109.47	0.093	0.043
**Down regulated**							
IL-7	18.74 ± 10.92	13.95 ± 4.99	0.312	16.29 ± 11.63	30.56 ± 12.67	0.049	0.029
ICAM-1	4,661.19 ± 1,351.69	4,444.89 ± 1,117.25	0.750	4,019.01 ± 1,637.88	5,025.26 ± 714.98	0.174	0.039
CCL-11	166.62 ± 132.93	154.34 ± 92.14	0.844	109.73 ± 64.04	180.09 ± 104.57	0.155	0.048

*BDNF, brain-derived neurotrophic factor; CCL, C-C motif chemokine ligand; ICAM, intercellular adhesion molecule; IL, interleukin; IVT, intravenous thrombolysis; SD, standard deviation; sICH, symptomatic intracranial hemorrhage; VEGF, vascular endothelial growth factor*.

*The value of p represented a comparison of concentration prior to and 24 h after IVT in each group.

*^†^The value of p represented a comparison of changed concentration prior to and 24 h after IVT between sICH and non-sICH*.

### Function Enrichment and Protein–Protein Interaction Network Analysis

**Figure 3:**
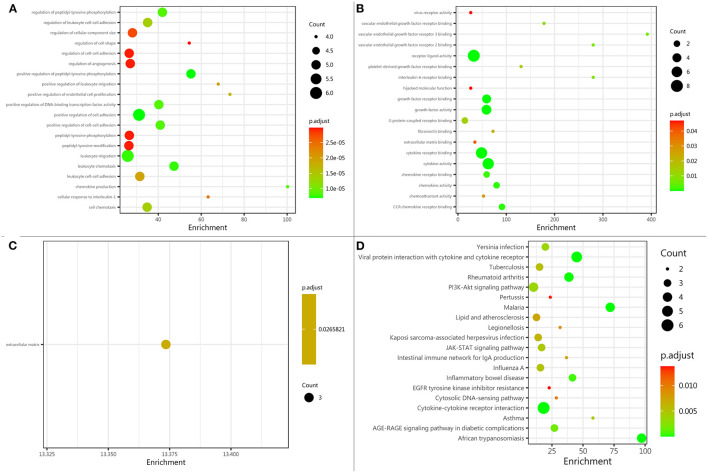
Protein function analysis of identified biomarkers. **(A)** Top 20 significantly enriched biological processes of identified biomarkers; the *X*-axis represents the enrichment, while the *Y*-axis represents the biological process. **(B)** The molecular function enriched by identified biomarkers; the *X*-axis represents the enrichment, while the *Y*-axis represents the molecular function. **(C)** The cellular component enriched by identified biomarkers; the *X*-axis represents the enrichment, while the *Y*-axis represents the cellular component. **(D)** The pathway enriched by identified biomarkers; the *X*-axis represents the enrichment, while the *Y*-axis represents the pathway. The deeper the color, the larger the value of *p*; the larger the circle, the bigger the counts.

A comprehensive gene ontology (GO) enrichment analysis was used to gain a deeper insight into the main functions of the identified biomarkers. The GO analysis consisted of biological processes, molecular functions, and cellular component analysis. The biological process analysis showed that ICAM-1, IL-6, IL-7, IL-10, IL-18, and VEGF were mostly included in the positive regulation of cell adhesion (Figure 3A). The molecular function analysis showed that CCL-11, CCL-24, IL-6, IL-7, IL-10, IL-18, and VEGF, were mostly included in the cytokine activity (Figure 3B). The cellular component analysis showed that IL-7, ICAM-A, and VEGF, were mostly included in the extracellular matrix (Figure 3C). The Kyoto Encyclopedia of Genes and Genomes (KEGG) enrichment analysis showed that CCL-11, CCL-24, IL-6, IL-7, IL-10, and IL-18, were included in cytokine–cytokine receptor interaction (Figure 3D).

**Figure 4:**
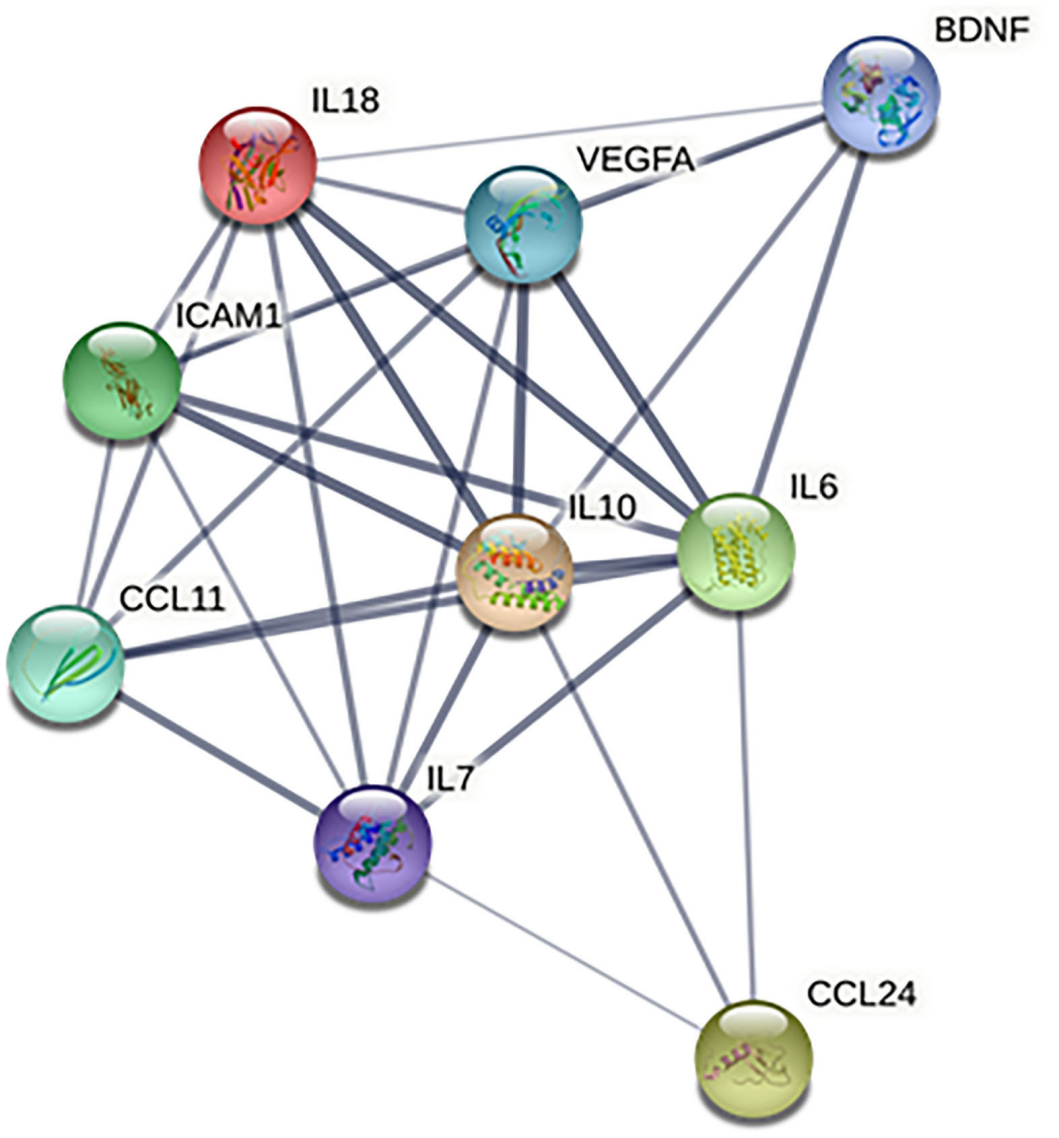
Protein–protein interaction network of identified biomarkers. BDNF, brain-derived neurotrophic factor; CCL-11, C-C motif chemokine ligand (CCL)-11; CCL-24, C-C motif chemokine ligand (CCL)-24; ICAM-1, intercellular adhesion molecule-1; IL-10, interleukin-10; IL-18, interleukin-18; IL-6, interleukin-6; IL-7, interleukin-7; and VEGF, vascular endothelial growth factor.

Based on the information from the Search Tool for the Retrieval of Interacting Genes (STRING) database, the protein–protein interaction network constructed by the above 9 identified biomarkers was obtained (Figure 4). The results showed that IL-10 and IL-6 (degree = 8) could interact with the most biomarkers, followed by IL-18, IL-7, and VEGF (degree = 7), ICAM-1 and CCL-11 (degree = 6), BDNF (degree = 4), and CCL-24 (degree = 3).

## Discussion

To the best of our knowledge, this is the first report that identified significant changes in 9 serum biomarkers in a sICH group compared with those in a non-sICH group, including increased biomarkers, such as BDNF, CCL-24, IL-6, IL-10, IL-18, and VEGF, and decreased biomarkers, such as CCL-11, ICAM-1, and IL-7.

There have been a lot of studies to investigate the association of biomarkers with post-thrombolytic hemorrhagic transformation in stroke (6–11). However, these studies have not investigated changes in serum biomarkers before and after sICH in thrombolytic stroke. Our results showed that these significantly changed biomarkers mainly contributed to the inflammatory response. As a result of recruited leukocytes and released proinflammatory cytokines, inflammation contributed to brain injury following ICH (16). Our results indicate that neuroinflammatory responses play an important role in post-thrombolytic sICH.

In the present study, we found significantly decreased circulating levels of IL-7, ICAM-1, and CCL-11 in the sICH patients, compared with non-sICH patients, which is consistent with previous studies (17–19). After stroke injury, peripheral leukocytes migrate into injured brain tissue through adhesion molecules and chemokines (20). As an adhesive protein, ICAM-1 facilitates leukocyte adhesion to vascular endothelium and entry into brain tissue through the blood-brain barrier (21–24). The CCL-11 is a proinflammatory cytokine and induces chemotaxis of leukocytes into the injured brain region (24). IL-7 mainly activates recruited T lymphocytes and further produces proinflammatory factors (25–27). As ICAM-1, CCL-11, and IL-7 were widely involved in transmigration and activation of leukocytes, we hypothesized that a decrease in the three biomarkers could be due to their significant consumption of biomarkers by migrating into injured brain tissue along with leucocytes after sICH. Additionally, tPA was reported to induce a surge of circulating immune cells and mobilize peripheral neutrophils and T cells (28), which may also be involved in the neuroinflammatory response.

Our study also found that CCL-24, IL-6, and IL-18 significantly increased after sICH, compared with patients with non-sICH. These biomarkers act as proinflammatory factors and express in the recruited leukocytes and activated glia. For example, CCL-24 was found to act directly on chemokine receptor CCR-3 to recruit leukocytes and play a role in mediating the neuroinflammation set off by ischemic-reperfusion injury (29, 30). IL-6 was produced in activated microglia and caused the brain injury after intracerebral hemorrhage under inflamed conditions (31, 32). IL-18, produced by activated macrophages and involved in neuroinflammation (33), was correlated with poor outcomes in intracerebral hemorrhage (34). However, as shown in Table 2, the concentration of IL-6 significantly increased after IVT in both the sICH and non-sICH groups, which was not consistent with the other two biomarkers. These results suggested that IL-6 may be triggered to secrete by ischemic or hemorrhagic brain injury, while CCL-24 and IL-18 were specifically related to sICH.

In addition, BDNF, IL-10, and VEGF were significantly increased after sICH, which was consistent with previous studies (35–37). BDNF, secreted from microglia, was found to be involved in the endogenous neuroprotection after brain injury (35, 38–40). As an anti-inflammatory cytokine, IL-10 is secreted mainly by the lymphocytes and monocytes and it inhibits transcription and production of the proinflammatory factor IL-6 (41, 42), which was reported to be associated with better neurological prognosis (43). Additionally, VEGF was an important angiogenesis regulatory factor and mainly enhanced vascular permeability and promoted endothelial cell proliferation after ICH (44). As shown in Table 2, the concentrations of IL-10 and VEGF increased in two groups, which was not consistent with BDNF. These results suggest that as a self-defending mechanism, the secretion of IL-10 and VEGF may be over-released from the glia and leukocytes in response to ischemic or hemorrhagic brain injury, while BDNF may be specifically triggered by hemorrhagic insult.

Unexpectedly, we did not detect significant changes in other preset biomarkers, such as MMP-9 and ferritin, which were previously reported to be associated with post-thrombolytic hemorrhagic transformation in ischemic stroke (6). The negative results may be attributed to the following reason. Most previous studies mainly detected the levels of biomarkers at admission (45, 46), while the present study focused on investigating the change from admission to 24 h later.

Based on functional enrichment analysis, cell adhesion positive regulation, cytokine activity, extracellular matrix, and cytokine–cytokine receptor interaction were significantly enriched items. After a stroke, adhesion molecules (e.g., ICAM-1) mediated several leukocytes and cytokines (e.g., VEGF and IL-7) entered into the brain through the injured blood-brain barrier (20, 22). The chemokines (e.g., CCL-11 and CCL-24) recruited leukocytes to mediate activation and release of proinflammatory cytokines (e.g., IL-6 and IL-18) (47). In addition, the cytokine–cytokine receptor interaction between CCL-24 and CCR-3 mediated the recruitment of leukocytes (29). Therefore, we inferred that cell adhesion regulation, cytokine activity, and cytokine–cytokine receptor interaction may contribute to brain injury after ICH through mediating recruitment of leukocytes and releasing of proinflammatory cytokines.

We acknowledge that our study has several limitations. First, it is a small-sample study with only 7 AIS patients with post-thrombolytic sICH in 24 h and collecting blood samples. Additionally, the study only collected blood samples before and 24 h after IVT. A large cohort study with samples collected at more time points (e.g., 2, 6, and 12 h after IVT) is warranted. Second, only serum biomarkers were measured in the present study. Given the neuroinflammation-related or neuroprotection-related biomarkers, it may provide valuable information if these biomarkers are measured in the brain such as CSF. Third, the sensitivity and specificity of identified biomarkers for predicting sICH need to be validated in the future. Fourth, the change of biomarkers in patients with different etiologies should be investigated in the future. Finally, although baseline data were matched through propensity scores matching, some potential biases may be inevitable. For example, there may exist an imbalance in the baseline circulating levels of biomarkers.

## Conclusion

For the first time, the present study prospectively investigated the changes of preset 49 serum biomarkers in patients with ischemic stroke receiving thrombolysis and found that 6 neuroinflammatory and 3 neuroprotective biomarkers were involved in the brain injury following post-thrombolytic sICH. The role of these biomarkers warrants further investigation given the potential therapeutic implications.

## Data Availability Statement

The raw data supporting the conclusions of this article will be made available by the authors, without undue reservation.

## Ethics Statement

The studies involving human participants were reviewed and approved by Institution Human Research Ethics Committees of General Hospital of Northern Theater Command. The patients/participants provided their written informed consent to participate in this study.

## Author Contributions

H-SC designed and reviewed. YC conducted the analyses and drafted the manuscript. X-HW, YZ, S-YC, B-YS, and L-HW contributed to the implementation of blood sample collection. All authors contributed to the article and approved the submitted version.

## Funding

The study was funded by grants from the National Key R&D Program of China (2017YFC1308203) and the Project on Research and Application of Effective Intervention Techniques for Chinese Stroke Guidelines from the National Health and Family Planning Commission in China (GN-2016R0008).

## Conflict of Interest

The authors declare that the research was conducted in the absence of any commercial or financial relationships that could be construed as a potential conflict of interest.

## Publisher's Note

All claims expressed in this article are solely those of the authors and do not necessarily represent those of their affiliated organizations, or those of the publisher, the editors and the reviewers. Any product that may be evaluated in this article, or claim that may be made by its manufacturer, is not guaranteed or endorsed by the publisher.
